# Green Tea and Its Relation to Human Gut Microbiome

**DOI:** 10.3390/molecules26133907

**Published:** 2021-06-26

**Authors:** Sergio Pérez-Burillo, Beatriz Navajas-Porras, Alicia López-Maldonado, Daniel Hinojosa-Nogueira, Silvia Pastoriza, José Ángel Rufián-Henares

**Affiliations:** 1 Departamento de Nutrición y Bromatología, Instituto de Nutrición y Tecnología de los Alimentos, Centro de Investigación Biomédica, Universidad de Granada, 18071 Granada, Spain; spburillo@ugr.es (S.P.-B.); beatriznavajas@ugr.es (B.N.-P.); alicialopez@ugr.es (A.L.-M.); dhinojosa@ugr.es (D.H.-N.); spdelacueva@ugr.es (S.P.); 2 Department of Biochemistry and Molecular Biology, Boonshoft School of Medicine, Wright State University, Dayton, OH 45435, USA; 3 Instituto de Investigación Biosanitaria ibs. GRANADA, Universidad de Granada, 18071 Granada, Spain

**Keywords:** green tea, gut microbiota, catechin, polyphenols, health

## Abstract

Green tea can influence the gut microbiota by either stimulating the growth of specific species or by hindering the development of detrimental ones. At the same time, gut bacteria can metabolize green tea compounds and produce smaller bioactive molecules. Accordingly, green tea benefits could be due to beneficial bacteria or to microbial bioactive metabolites. Therefore, the gut microbiota is likely to act as middle man for, at least, some of the green tea benefits on health. Many health promoting effects of green tea seems to be related to the inter-relation between green tea and gut microbiota. Green tea has proven to be able to correct the microbial dysbiosis that appears during several conditions such as obesity or cancer. On the other hand, tea compounds influence the growth of bacterial species involved in inflammatory processes such as the release of LPS or the modulation of IL production; thus, influencing the development of different chronic diseases. There are many studies trying to link either green tea or green tea phenolic compounds to health benefits via gut microbiota. In this review, we tried to summarize the most recent research in the area.

## 1. Introduction

Tea is a beverage that is thousands of years old and has not lost its popularity due to different health benefits, pleasantness, or social characteristics [1,2,3]. Made from the infusion of the leaves of the *Camellia sinensis* plant, tea has been used in traditional Chinese medicine for over 3000 years [2,4]. Tea has expanded over the centuries from China throughout the rest of Asia and then to Europe, America, and finally Africa [2]. This expansion was made possible through colonization and different trade routes such as the silk road [2]. Tea is, therefore, one of the most consumed beverages around the world, whose popularity has recently increased due to the extensive research claiming associations between tea and health [2].

According to the processing treatments of the leaves, the enzymatic changes and fermentation processes, we can find different kinds of tea, black tea, dark tea, yellow tea, oolong tea, green tea, and white tea. [5]. This review will focus on green tea. To produce green tea, its leaves are subjected to different processes such as steaming at high temperatures or roasting, which causes the inactivation of the oxidative enzyme polyphenol oxidase. [5]. The inactivation of polyphenol oxidase is what prevents most of its polyphenolic compounds from being oxidized (catechins, flavones, anthocyanins, and phenolic acids; and some other minor polyphenols that also exist, such as epigallocatechin gallate (EGCG), flavanol glycoside, and tannins) [5]. 

Over the years, tea along with its antioxidant and anti-inflammatory properties has been associated to multiple health benefits, namely in the treatment of obesity, diabetes, cancer, kidney, liver, brain and bone diseases, among others [2]. On the other hand, some of these health benefits have been attributed to an inter-play between tea bioactive compounds and the gut microbiota [6]. As it has been extensively demonstrated during the past decade, tea polyphenols have low bioavailability, most of them reaching the large intestine where they are metabolized by gut microbes [7]. Health benefits derived from tea-microbiota inter-play could result from direct effect of microbial polyphenol metabolites or from an indirect effect derived from stimulation of specific beneficial gut microbes [7]. At the same time, gut microbiota is also closely related to the host health and play important roles in chronic diseases such as obesity, diabetes, inflammatory bowel disease, or even neurological disorders [8,9]. Therefore, much efforts are being spent on trying to understand how gut microbiota specifically works, especially in relation to diet [10].

Since tea is one of the most consumed beverages around the world and has potential to influence gut microbes due to its content in polyphenols, many studies can be found about this theme. In this review, we aimed to gather the most recent research that associates green tea or green tea bioactive components with gut microbiota and specific health benefits. 

## 2. Phenolic Content of Green Tea and Their Relation with Gut Microbiota. Gut Bacteria Genera and Species Stimulated or Inhibited by Green Tea or Its Components

Phenolic compounds constitute 24–36% of the dry weight of green tea, followed by protein content (15%), lignin (7%), amino acids (3–4%), caffeine (2–4%), organic acids (2%), and chlorophyll (0.5%). [4]. Green tea’s most abundant polyphenols are catechins, representing around 30–40% of the tea leave dry weight [11]. Catechins belong to the polyphenol family of flavan-3-ols and in green tea they can be found in several forms being the most abundant (−)-epicatechin (EC), (−)-epigallocatechin (EGC), (−)-epicatechin-3-gallate (ECG), and (−)-epigallocatechin-3-*O*-gallate (EGCG). [12]. On the other hand, fermented teas (such as black and red) have also high concentrations of theaflavins, which are formed via catechin oxidation during fermentation [11]. However, in this review, we focused on catechins since they are the main components of green tea.

Tea catechins can favor the growth of potentially beneficial bacteria, though at the same time they can also hinder that of some potentially detrimental microbes [13]. Hence, polyphenols were included among the different substances that could act as prebiotics [14]. Authors have proposed that the stimulating effect that tea polyphenols could have on specific gut microbes could be due to the ability of such microbes to use and metabolize specific phenolics compounds [7]. On the other hand, phenolic compounds are secondary plant metabolites whose main function is to protect plants against certain aggressions or infections either by bacteria, insects, etc. [7]. Therefore, tea phenolic compounds (i.e., catechins) have also shown inhibitory effects on some bacteria such as *Bacillus cereus, Campylobacter jejuni, Clostridium perfringens, Escherichia coli, Helicobacter pylori, Legionella pneumophila, and Mycobacterium spp.* [7]. This inhibitory effect has been attributed a disruption of the cell membrane that is caused by such polyphenols. For instance, it has been observed how EGCG, can bind with peptidoglycans from cell membranes in gram positive bacteria and cause a disruption. Gram negative bacteria are therefore protected against such inhibitory mechanism thanks to the outer membrane and the negatively charged lipopolysaccharide that repels catechins [15]. However, some gram-positive bacteria are not negatively affected either, which suggests another mechanism involved though it is not clear.

There are many studies where green tea, or some of its catechins, have shown that they stimulate and/or hinder the growth of specific gut bacterial species. Zhang et al. [16] found in vitro that EGCG, gallocatechin gallate (GCG) and EGCG 3”-methyl could significantly increase the abundance of *Bifidobacterium spp., Lactobacillus,* and *Enterococcus*, and increase the production of short chain fatty acids (SCFA), the main microbial fermentation product, main energy source for colonocytes, and is beneficial for human health [8]. In the same study, they found that these catechins were also able to hinder the growth of *Bacteroides, Prevotella, Clostridium hystoliticum, Eubacterium,* and *Clostridium* group [16]. However, in another in vitro batch fermentation study, it was found that epicatechin was able to promote the growth of *Eubacterium rectale* and *Clostridium coccoides* [17]. These authors acknowledged that though catechin and epicatechin showed potential to shape gut microbiota community structure, their batch culture was not enough evidence and in vivo investigations were needed. The *Bacteroidetes* phyla was also found to be increased in response to green tea polyphenols in an in vitro human colonic model [18]. *Firmicutes*, on the other hand, has been usually found to be decreased as consequence of green tea dosing, which could be explained, in part, by the inhibitory effect of polyphenols over gram positive bacteria. Liu et al. [19] in vitro fermented EGCG to monitor its degradation by gut microbes. They found nine different operational taxonomic units (OTUs) that were significantly more abundant in EGCG treated samples and 28 OTUs that apparently were inhibited by EGCG. Among the former, there were five *Bacteroides* species and *Clostridium symbiosum, Christensenellaceae, Ruminococcus bromii,* and *Bifidobacterium adolescentis* [19]. *C. symbiosum* is a SCFA butyrate-producing bacterium, and this way is associated with beneficial health effects. The *Christensenellaceae* family has also been associated with important beneficial effects such as lower BMI and increased longevity [19]. Conversely, the effects of *R. bromii* on human health are not clear yet (although it plays an important role in starch degradation), unlike the beneficial effects of Bifidobacteria on human health, which are widely known, constituting an important probiotic [8,19]. On the other hand, they found how EGCG inhibited *Forsterygion varium* which, according to these authors, could prompt pro-inflammatory responses in the gut. Other inhibited bacteria found in this study were *Bilophila* and the family *Enterobacteriaceae*, which are related to gastrointestinal disorders [19].

Several other studies carried out in mice found how green tea extracts favored the growth of *Akkermansia,* a genus known to degrade mucin [20,21]. EGCG was found to favor the growth of *Akkermansia* though, at the same time, this catechin reduced the abundance of the phyla *Firmicutes* and the overall alpha diversity of the community. Alpha diversity refers to a measure of microbiome diversity in a single sample [21]. This was explained by the antibacterial activity of EGCG [21]. *Akkermansia* has been given some importance due to its apparent correlation with lipid metabolism and obesity, being even considered as an anti-obesogenic bacterium [22,23,24]. The abundance of this microbe was found to be correlated with a reduced accumulation of saturated fatty acids in the plasma of mice, although confirmation is needed [22]. On another study, rats were dosed with green tea polyphenols at different concentrations, finding that green tea polyphenols decreased diversity and shaped microbial communities in a dose-dependent manner [25]. These authors found that the phyla *Bacteroidetes* also increased in a dose-dependent manner in rats fed with green tea polyphenols. *Bacteroidetes* includes many bacteria in charge of fiber degradation and it has also been found to be depleted in obese mice and humans and to be correlated with body weight loss [25]. Moreover, an OTU belonging to the genus *Oscillospira* was found to be increased in response to green tea polyphenols intake. This genus has been previously related to lean people, though more research is needed in that regard [26].

However, there are some important contradictory results. In a human intervention where healthy volunteers were given green tea, authors found that alpha diversity was increased and associated with tea drinking [27]. In addition, they found an increase in *Firmicutes* and a decrease in *Bacteroidetes*, which goes in opposite direction to what other studies have found. On the contrary, an increase in SCFA producing bacteria was also found, which coincides with other investigations. Among these genera there were *Faecalibacterium, Roseburia, Blautia, Eubacterium, Bifidobacterium*, and *Coprococcus* [27].

Moreover, Janssens et al. [28] carried out a human intervention that lasted 12 weeks, in which 58 subjects were either given or not given green tea. These authors did not find any significant differences between the gut microbiome at baseline and after 12 weeks of tea drinking. However, phylogenetic information was only provided at the phylum level and maybe some differences could still be observed at a lower level. Regardless, results found in this study are different than those found in either in vitro or in vivo that claim dramatic changes in the gut microbiome. All of these contradictory results are showing how difficult it is to study the gut microbiome and to carry out reproducible research.

Alteration of the gut microbiota community structure by green tea polyphenols also imply an alteration of the metabolic pathways carried out by the microbial community [18]. In this regard, Zhou et al. [29] demonstrated how several metabolic pathways were altered in rats after a 6-month treatment with green tea polyphenols. Specifically, they observed a decrease in carbohydrate energy scavenging pathways, a decrease in bile acid synthesis pathways, fatty acids absorption, and a higher production of hexoses and vitamins, along with an altered metabolism of amino acids. These metabolic changes in the gut could have important repercussions in the host health, for instance in relation to obesity, due to a decrease in energy scavenging [29]. In another study, Zhou et al. [30] found that there was a connection between the gut microbiota’s tricarboxylic acid cycle (TCA) and urea cycles and the macronutrient metabolism in the host (in this study rats). Therefore, TCA cycle in the gut microbiota is closely related to systemic energy utilization in the host (mitochondrial TCA), which could be improved by green tea polyphenols.

## 3. Metabolism of Tea Phenolic Compounds by Gut Microbiota

The metabolism of phenolic compounds by gut microbes has been extensively studied since many of their health benefits are, in fact, attributed to microbial metabolites [11]. It is currently estimated that only between 10 and 20% of dietary polyphenols are absorbed in the small intestine [31]. Therefore, most of them reach the colon and are metabolized by gut microbes into smaller compounds [8].

**Figure 1:**
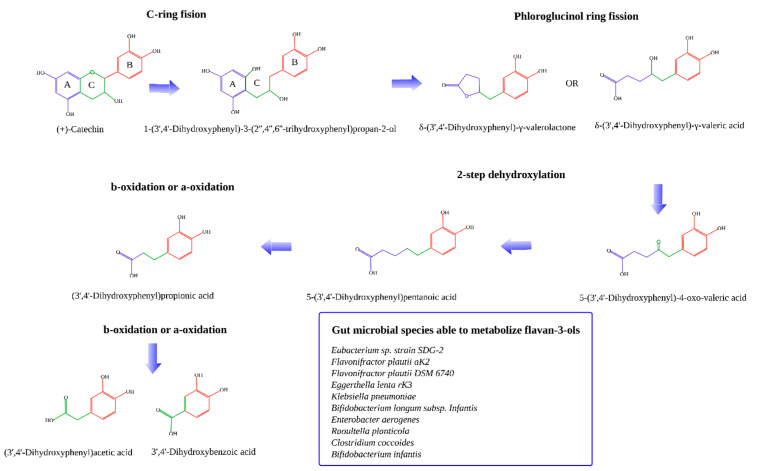
Metabolization of flavan-3-ols by the gut microbiota [7,8,11,32,33,34].

Gut microbial degradation of all different forms of catechins have been extensively detailed before [7,8,11,32,33,34]. Briefly, microbial degradation of catechins starts with the cleavage of the C ring followed by dehydroxylation of some of the positions of the B ring. After that, the A ring (phloroglucinol ring) is cleaved forming the corresponding hydroxy-phenylvaleric acids and phenyl-gamma-valerolactones [35]. These can then be dehydroxylated to form the corresponding valeric acids. Afterwards, these suffer beta or alpha-oxidation to finally obtain smaller phenolic acids: 3-hydroxybenzoic acid, 4-hydroxybenzoic acid, 3-hidroxyphenylpropionic acid, and 4-hydroxyphenylpropionic acid (Figure 1). Phloroglucinol could also be a final product [32].

The gut bacterial species identified to be able to metabolize flavan-3-ols (i.e., catechins) are: *Eubacterium* sp. strain SDG-2, *Flavonifractor plautii* aK2, *Flavonifractor plautii* DSM 6740, *Eggerthella lenta* rK3, *Klebsiella pneumoniae*, *Bifidobacterium longum* sp. *Infantis, Enterobacter aerogenes, Raoultella planticola, Clostridium coccoides,* and *Bifidobacterium infantis* [8,11]. 

Additionally, hydroxyphenyl-gamma-valerolactones have been also reported as metabolites. They are found in urine in their glucuronidated and sulfated forms after being processed in the liver [33]. In fact, the metabolite 5-hydroxyphenyl-gamma-valerolactone is able to pass through the blood–brain barrier, which has been related to the potential neuroprotective effect of foods rich in phenolic compounds [36]. Finally, the metabolites 3-hydroxybenzoic acid and 3-hydroxyphenylpropionic acid were found to accumulate in the brain and hinder the appearance of neurotoxic beta-amyloid aggregates related to Alzheimer’s disease [33]. Both of them highlight the importance of the “gut–brain axis”. 

However, it has also been demonstrated that antibiotic treatment can alter catechin metabolization by gut microbes [37]. According to these authors, mice treated with antibiotics showed higher levels of EGCG in blood, liver, and urine, probably due to an elimination of microbes in charge of catechin metabolization. Therefore, catechins bioavailability will be determined, at least in part, by the gut microbiota.

## 4. Influence of Green Tea on Health via Gut Microbiota

**Figure 2:**
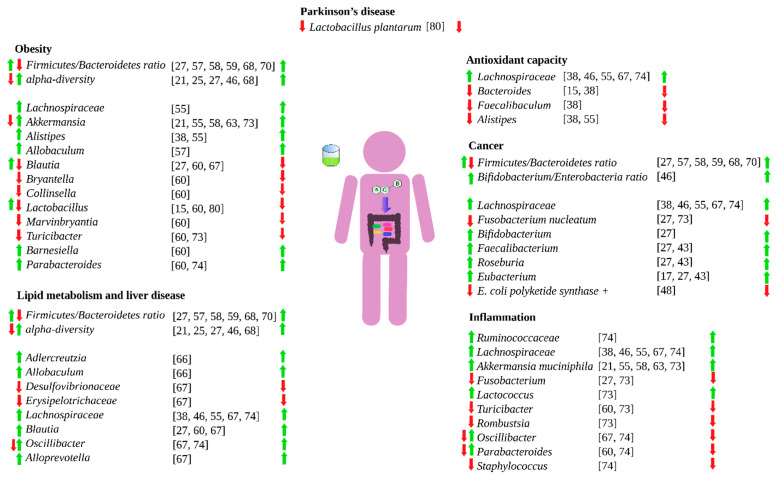
Influence of green tea on different health aspects via gut microbiota. Left-hand arrows means that green tea either favors (green) or hinder (red) the specific bacteria growth. Right-hand arrows means that the bacteria effect is either beneficial (green) or detrimental (red).

Green tea has extensively demonstrated an influence human health [2]. However, green tea can also do so by shaping and modulate the human gut microbiota [6]. Green tea can favor the growth of beneficial bacteria, inhibit the growth of detrimental ones, or increase the production of beneficial metabolites such as short chain fatty acids. By shaping and modulating the gut microbiota, green tea can influence gut inflammatory processes, colorectal cancer, redox processes in the intestine, energy scavenging, macronutrient metabolism by gut microbes, and obesity. Figure 2 summarizes how green tea can influence the human health via gut microbiota.

### 4.1. Antioxidant Capacity, Scavenging Capacity and Their Relation with Gut Microbiota

Oxidative stress has been related to a number of chronic diseases. Polyphenols, including green tea polyphenols, are considered potent antioxidants and free radical scavengers [4]. Hence, their attributed beneficial role against oxidative stress-related chronic diseases. 

In an in vitro fermentation study using rat fecal material, Chen et al. [35] demonstrated how the metabolization of catechins by gut microbes actually changes the antioxidant activity of the original molecules. They observed how the first metabolite obtained after the cleavage of the C ring was actually the most antioxidant, two-times higher than the original compound. At the same time, dehydroxylation reactions greatly decreased antioxidant activity. Final metabolic products, such as phenylpropionic acids, still conserve some antioxidant activity, but lower than that of the original catechins. Additionally, due to their ability to capture free radicals, green tea polyphenols are able to influence the gut microbial TCA, which, at the same time can, influence the energy balance or its utilization in the host [30].

On the other hand, Ma et al. [38] carried out a study in which mice were fed different doses of tea polyphenols. They found that reactive oxygen species levels and those of superoxide dismutase (SOD) and glutathione (GSH) were significantly related to gut microbiota community structure. Whereas, unidentified *Lachnospiraceae* was positively correlated (Spearman) with SOD and GSH and negatively with ROS, *Bacteroides, Faecalibaculum,* and *Alistipes* showed opposite relationships. These authors suggested that the redox state of the gut could be associated with its bacterial community. 

Besides reactive oxygen species, there are other toxic waste metabolites that accumulates during cellular metabolism, such as ammonia and reactive carbonyl species (RCS) [39]. Ammonia is mainly produced in the gut as a waste product from urea metabolism by gut bacteria [40]. The accumulation of both RCS and ammonia has been related to chronic diseases [40,41]. Zhang et al. [39] demonstrated in mice how the gut microbiota facilitates the formation of EGCG-Ammonia conjugates reducing its toxic effects. Additionally, they observed how EGCG as well as its amminated conjugate could bind RCS (such malondialdehyde or methylglyoxal) and therefore avoid their accumulation. As a possible explanation, these authors hypothesized that EGCG could be oxidized by gut microbes generating the corresponding quinone, which in turn would quickly react with ammonia.

### 4.2. Cancer

#### 4.2.1. Colorectal Cancer

SCFA producing bacteria have been related to the prevention of colorectal cancer [42]. Colorectal cancer patients have an increased inflammatory state in the intestine along with an overproduction of lipopolysaccharide (acts as an endotoxin promoting the release of pro-inflammatory citokynes) compared with healthy people [27]. Additionally, some gut bacterial species have been identified as potentially linked to colorectal cancer, such as *Helicobacter pylori, Bacteroides fragilis,* or *Fusobacterium nucleatum* [43]. However, not only specific bacterial species but also dysbiosis has been related to colorectal cancer. Dysbiosis is characterized by a decrease in the *Firmicutes/Bacteroidetes* ratio, which leads to a lower production of short chain fatty acids [43]. At the same time, SCFA have anti-inflammatory properties and positive effects on the immune system [44]. Therefore, it has been proposed that a decrease in SCFA producing bacteria and an increase in lipopolysaccharide producing species is related to colorectal cancer development. Some bacteria, such as *Bifidobacterium*, are able to provide SCFA and suppress LPS formation, creating therefore an anti-inflammatory environment [27]. Several studies described how either green tea polyphenols or green tea are able to increase SCFA production and to favor *Bifidobacterium* [27], *Faecalibacterium, Roseburia, Eubacterium,* and other SCFA producers [43].

Another possible cause for increased inflammation in the gut is the overgrowth of bacteria from the oral cavity. Therefore, bacteria that provides colonization resistance could help reduce inflammation [45]. Green tea consumption provides colonization resistance in a human trial by increasing the ratio *Bifidobacterium/Enterobacteria*, which is a well-known marker for colonization resistance [46]. Additionally, these authors found how *Lachnospiraceae* was also increased after tea consumption, which has been also associated with colonization resistance. *Fusobacterium*, a genus strongly associated with colorectal cancer [47], was also reduced by green tea drinking [27].

According to Yuan et al. [27] colorectal cancer can also be favored by uncontrolled biofilm formation in the gut. These types of biofilms appear with lower *Firmicutes/Bacteroidetes* ratios, which green tea polyphenols have shown to correct in healthy human volunteers and, thus, reducing biofilm formation. 

Another possible association between gut microbiota and colorectal cancer is the production of colibactin [48]. This is a genotoxic secondary metabolite produced by some gut bacteria, specifically those that have the polyketide synthase genomic island [48]. Among these bacterial species there are some *E. coli* strains. In an observational study carried out in Japan with adults, *E. coli* strains that carried the polyketide synthase genomic island were negatively associated with green tea intake [48]. However, there were more associations found and as the authors claimed, more studies are needed to validate such findings.

Finally, in an in vitro study, gut microbial metabolites of green tea polyphenols (phenylacetic acids and hydroxyphenyl valerolactones) have also proven to exert antiproliferative activity in HCT-116 cells [49]. In fact, these metabolites showed higher activity than that of the parent compound (EGCG). Therefore, not only parent compounds (such as EGCG) have been associated with cancer protection but also their metabolites, which raises the question of how the microbial metabolization of polyphenols could affect cancer prevention. This question was raised by Adami et al. [50], claiming that inter-individual variations in the microbial communities could lead to different responses against cancer. If a metabolite is an effective protector, people lacking the species in charge of producing it would potentially have less protection. This also works in the opposite way: EGCG has been linked to protective effects but if it is metabolized, then such potential effects could not occur. Furthermore, this question is applicable to any other activity attributed to phenolic metabolites but, specially to parent compounds, since they have been probably more studied, at least in vitro.

#### 4.2.2. Mammary Cancer

Sharma et al. [51] investigated the protective effect of green tea polyphenols, broccoli sprouts, and both together in the development of mammary cancer. For that purpose, the researchers used Her2/neu transgenic spontaneous ER(-) mammary cancer mouse models. These authors found that green tea polyphenols did not generate a significant change in gut microbiota community structure in healthy mice. However, green tea polyphenols did decrease Firmicutes relative abundance in a significant manner. Contrary to results found in healthy mice, green tea polyphenols did change the gut microbial community structure of mice after the tumor onset. Green tea polyphenols prompted a decrease in Proteobacteria and an increase in Bacteroides in comparison with control diet. More specifically, green tea polyphenols increased the abundance of Adlercreutzia, Lactobacillus, and Prevotella. Their conclusion was that that the combination of broccoli sprouts and green tea polyphenols was more protective than either of them separately. They also concluded that though green tea polyphenols did not show the most protective effect, they help preventing large alterations in the microbial composition due to the appearance of the tumor.

### 4.3. Obesity

Obesity is strongly related to different chronic diseases such as diabetes type II, cardiovascular diseases, metabolic syndrome, or some cancers. Therefore, many strategies have been developed to reduce obesity prevalence. Gut microbiota has proven to be intimately related to obesity [52]. These authors found that the *Bacteroidetes* phylum was significantly decreased in obese mice whereas phylum *Firmicutes* was significantly increased. Obese mice were more efficient at harvesting energy from substrates and therefore contributing to being overweight, but they also produced higher SCFA concentrations [53].

Green tea has been reported to protect against obesity via different mechanisms, such as interfering with lipid or carbohydrate metabolism, but also via modulation of the gut microbiota [54]. These claims are usually supported by the ability of tea to favor bacteria negatively associated with obesity and inhibit those positively linked. Liu et al. [55] found that oral administration of green tea to high-fat-diet induced obese C57BL/6J mice improved the gut microbial diversity. Additionally, green tea administration was able to shape gut microbial community structure, increasing the relative abundance of some beneficial (associated negatively with obesity) bacteria such as *Alistipes, Lachnospiraceae,* or *Akkermansia* [55]. In another study [56], green tea consumption was associated with a decrease in body weight and an improvement in metabolic syndrome symptoms, possibly decreasing LPS production and increasing SCFA. These results were found after feeding C57BL/6J mice with a high-fat diet added with tea water extract. Seo et al. [57] fed hamsters with a green tea obtained after being fermented by *Bacillus*. This extract was able to decrease the *Firmicutes* abundance and show possible anti-obesogenic effect by decreasing the energy harvested from macronutrients. Additionally, these authors found *Allobaculum* to be increased after tea intake. This abundance of this bacterium is higher after exercise in rats, and positively related to fiber intake and to healthy metabolic markers [57]. In another study, Remely et al. [58] fed obese mice with either a high-fat diet added or not with EGCG. These authors also found a higher *Firmicutes/Bacteroidetes* ratio in obese mice that was significantly corrected with the addition of EGCG to the diet. Similarly, Gou et al. [59] performed an experiment in which obese mice were fed with green tea polyphenols for three weeks. As other authors stated before, they found that the *Bacteroidetes* phylum increased with tea consumption, whereas the *Firmicutes* phylum decreased. Henning et al. [60] found, in addition to an increase in the *Firmicutes/Bacteroidetes* ratio, that green tea polyphenols stimulated the growth of different bacteria genera in diet-induced obese mice: *Blautia, Bryantella, Collinsella, Lactobacillus, Marvinbryantia, Turicibacter, Barnesiella*, and *Parabacteroides*. In addition, while *Barnesiella* and *Parabacteroides* were found to be negatively correlated (*p* < 0.05) to body weight, the other were positively correlated (*p* < 0.05). Whether these correlations have or do not have biological significance remains unclear. Similar results have been found in other studies, where *Firmicutes* was reduced by green tea polyphenols or by EGCG specifically [21,61,62].

Green tea and EGCG have been reported to increase the relative abundance of *Akkermansia muciniphila* in mice fed with high-fat diet [21,63]. However, this microbe has also been reported to decrease after supplementing a normal diet with EGCG [58], existing therefore certain discrepancies. Regardless, *A. muciniphila* has been linked to multiple health benefits in several pathological situations such as diabetes type II, obesity, or lipid metabolism alterations [22,24,64]. However, Zhu et al. [65] fed mice with green tea and did not observe significant changes in *A. miciniphila*. They did find that green tea consumption significantly decreased *Lactobacillus reuteri*, frequently found in the gut microbiome of obese people.

### 4.4. Lipid Metabolism and Hepatic Disease

It has been suggested that the gut microbial dysbiosis caused by high-fat diets may result in an alteration in bile acids metabolism. This alteration is related to the steatosis pathology seen in non-alcoholic fatty liver disease [66]. Ushiroda et al. [66] fed male C57BL/6N mice with EGCG, finding a global improvement in lipid metabolism and fatty lesions in the liver. They also found higher abundances of *A. muciniphila*, *Adlercreutzia,* and *Allobaculum* and a lower abundance of *Desulfovibrionaceae*, which were found to be related to an improvement in the concentration of bile acid conjugates in blood [66]. These results suggested a mechanism of action involving the gut microbiota and intestinal health. Similar results were found in another study in which mice were fed with high-fat diets with addition (or not) of green tea powder [67]. This study also describes that *Desulfovibrio* and *Erysipelotrichaceae* were significantly more abundant when green tea was not added. Some *Desulfovibrio* species, as well as the *Erysipelotrichaceae* family, have been related to inflammatory processes in the gastrointestinal tract and, in fact, *Desulfovibrio* is a potential LPS producer [67]. On the other hand, *Allobaculum*, and *Lachnospiraceae* were more abundant when mice were fed green tea powder. Although these were the most discriminant bacteria between high-fat diet and high-fat diet with added green tea, other bacteria also showed significant differences: *Oscillibacter, Ruminiclostridium, Butyrivibrio, Alloprevotella,* and *Blautia* were significantly increased when green tea was administered. 

The *Lachnospiraceae* family include some butyrate producing genera such as *Roseburia, Coprococcus,* or *Butyrivibrio* [44]. According to Wang et al. [67], *Blautia, Oscillibacter,* and *Alloprevotella* were negatively correlated with body weight and they improved lipid and carbohydrate metabolism; *Akkermansia* did not show any statistically significant differences though. According to these authors, green tea powder administration increased in a statistically significant manner in some pathways (KEGG pathways): lipid metabolism, fatty acid metabolism, butanoate metabolism, carbohydrate metabolism, or energy metabolism. At the same time, lipolysaccharide biosynthesis was decreased [67]. Wang et al. [68] also found how tea polyphenols were able to reduce the *Firmicutes/Bacteroidetes* ratio and increase community diversity in C57BL/6J mice fed with high-fat diet. These authors proposed that such a shift in gut microbiota could be related to body weight control and improvement in plasmatic lipid levels observed after 8 weeks of tea polyphenol treatment.

Dey et al. [69] fed C57BL/6J mice with either high-fat diet or low-fat diet and added catechin, EGCG, or green tea extract. The idea was to trigger nonalcoholic steatohepatitis and study whether green tea polyphenols would exert any protection. They observed how both polyphenols and green tea extract shaped gut microbiota and its functionality at the same time, and either of the treatments were able to protect reducing inflammation. However, they noticed that each treatment had a different effect on the gut microbiota, suggesting that the benefits observed in the liver were not, at least entirely, dependent on the gut microbiota [69]. Conversely, Ning et al. [70] suggested that shifts caused in gut microbiome by EGCG could be related to benefits against nonalcoholic steatohepatitis. They fed C57BL/6J mice with EGCG, observing a change in the microbial community similar to those described before and characterized by a decrease in *Firmicutes* and an increase in *Bacteroidetes*. Additionally, they found that several enzymes implicated in lipid accumulation were expressed in lower abundance when mice were fed with EGCG [70]. These authors therefore suggested a potential benefit against liver disease, though they acknowledged that more research was needed since results were not definitive. 

All of this information taken together suggests that either green tea or its phenolic components (or both) are able to counteract the gut microbial dysbiosis originated by high-fat diets and potentially improve lipid metabolism.

### 4.5. Inflammatory Bowel Disease

The gut microbiota has been extensively related to gut inflammation [71,72] and gut microbial dysbiosis seems to be closely related to inflammatory bowel disease, including ulcerative colitis [73]. For instance, these patients have low alpha diversity indexes and high abundances of potentially inflammatory bacteria such as *Enterobacteriaceae* and *Fusobacteriacea* [71]. In a study in which mice were fed with green tea [73], gut microbiota was positively modulated. Microbiota from mice fed with either green tea or normal diet was transplanted to mice with chemically induced colitis; potentially beneficial bacteria, such as *Akkermansia* or *Lactococcus*, were found in higher abundances in mice that received fecal transplantation from green tea fed mice, whereas other potentially pathogenic bacteria were reduced (*Turicibacter* or *Rombustsia*) [73]. Fecal transplantation of microbiota from green tea treatment resulted in less intestinal inflammation and tissue damage. However, green tea intake did not down-regulate the NLRP3/ASC/caspase-1 pathway (this system is triggered by pathogens or damage and results in the maturation of the pro-inflammatory cytokine IL1b). On the other hand, green tea treatment did down-regulate another inflammatory pathway: TLR4/MyD88/NF-κB [66]. TLR4 is a transmembrane protein involve in the immune system that activates as a response to pathogens or molecular signs of damage. This protein binds with LPS and then via MyD88 activates NF-kB which triggers pro-inflammatory cytokine production [73]. Overall, fecal transplantation from green tea fed mice resulted in a positive modulation of gut microbiota and a reduction of the inflammation.

In another study, green tea treatment significantly improved the dysbiosis generated by colitis in mice [74]. Additionally, green tea treatment reduced the abundance of some bacteria that has been related to inflammation, such as *Bacteroides*. Another down-regulated bacterium was *Oscillibacter*, which, according to the bibliography, has been related to IL-10 production or intestinal dysbiosis in high-fat diets [74]. Moreover, *Parabacteroides* and *Staphylococcus* were also inhibited by green tea treatment. These bacteria are correlated to intestinal inflammation [74].

SCFA have been related to the intestinal health thanks to their anti-inflammatory properties among others [75]. Green tea treatment of male Fischer rats resulted in a stimulation of some butyrate producing bacteria from the family *Ruminococcaceae* and *Lachnospiraceae*. One possible explanation suggested by these authors is the biotransformation of green tea polyphenols by gut microbes. On the one hand, certain species are stimulated or favored by specific phenolics. On the other hand, phenolic metabolites could have a better bioavailability and bioactivity than the parent compounds, ranging from antioxidant activity to anti-inflammatory capacity as previously suggested [76].

On another note, L-theanine, an amino acid present in tea, was also studied regarding its potential anti-inflammatory properties and relation with gut microbiota in mice with chemically induced colitis [77]. However, though L-theanine demonstrated having anti-inflammatory properties, it was unable to revert the dysbiosis generated by colitis in the same way green tea demonstrated before [66,67].

### 4.6. Bone Health

Previous research has showed how gut microbiota is altered in osteoporotic or osteopenic people [78]. Specifically, *Firmicutes* are usually abnormally increased while *Bacteroidetes* are significantly decreased. This information suggests that the gut microbiota could play a role in bone health maintenance. Elmassry et al. [79] carried out a study using obese mice to investigate whether green tea extracts had or not any positive effect on bone density and whether this was in fact related to the gut microbiota. These authors found that green tea supplementation improved bone health by increasing the levels of collagen and bone volume of the femur and the LV-4 vertebra. Additionally, green tea intake shaped gut microbial communities in favor of some potentially beneficial microbes, such as *Akkermansia* and some others included in the *Ruminococcaceae* family. Finally, they found that green tea increased microbial pathways related to vitamin K2 biosynthesis. These authors concluded that green tea could improve bone health or have osteoprotective effects that were mediated via gut microbiota [79]. However, they actually did not provide any proof of such relation besides both bone density markers and gut microbiota changes happening at the same time. That is, both could be unrelated.

### 4.7. Parkinson’s Disease

Although its etiology is extremely complex and partially unknown, Parkinson’s disease is still the second most common neurodegenerative disease in humans [80]. Regardless, most risk factors are thought to be genetic and environmental [80]. Most cases of Parkinson’s disease are sporadic ones, but there is a small percentage of cases that are due to mutations of PTEN-induced kinase 1 (PINK1), a mitochondrial kinase. On the other hand, there is an ever more growing notion that Parkinson’s disease, as well as many other neurodegenerative alterations, are related to the gut microbiota via what is called the brain–gut axis [9,80]. At the same time, *Drosophila melanogaster* has proven to serve as a model due to how easy it is to genetically manipulate it. Taking this information into account, D. *melanogaster mutants* (PINK1 mutants) were administered EGCG showing an improvement in neurological symptoms [80]. Additionally, EGCG restored up to a certain point gut microbial alteration that appeared as consequence of the Parkinson’s disease [80]. More interestingly, symptoms worsened when antibiotics were administered [80]. They found that the genera *Lactobacillus* and *Acetobacter* were decreased after EGCG intervention [80]. *Lactobacillus plantarum*, *Acetobacter pomorum*, *Lactobacillus brevis,* and *Acetobacter pasteurianus* were identified as the species present in the gut of the flies [80]. Further experiments demonstrated that only *L. plantarum* and *A. pomorum* were really inhibited by EGCG [80]. However, *L. plantarum* demonstrated to exacerbate Parkinson’s disease symptoms [80]. The same authors discovered, via transcriptomic analysis, that the TotM gene was the main responder to EGCG and that the removal of the gene resulted in a blockage of the improvements [80]. Therefore, they concluded that the pathway gut microbiota-TotM had to play an important role in the neuro-protection exerted by EGCG [80].

### 4.8. Circadian Rhythm

The circadian rhythm is an internal process, mostly regulated from the hypothalamus, that regulates the day and night cycle every 24 h. It heavily influences physiological and metabolic processes, being this way significantly related to our health and wellbeing [81,82]. Disruption of this rhythm can be detrimental and lead to, among other diseases, metabolic syndrome, cancer, or cardiovascular disease. The gut microbiota is possibly affected by day and night cycles [82]. Intestinal microbiota has been reported to participate in a variety of physiological response like nutrients absorption, energy modulation, or the regulation of immune system. All of these are processes that are characterized by a dominant diurnal rhythm [62]. Also, the disruption of the circadian system can alter microbiome communities affecting the host metabolism in this way; for example, by perturbing energy homeostasis and inflammatory pathways, which leads to the metabolic syndrome [82]. Related to green tea, Zhang et al. [62] carried out an investigation in which the circadian rhythm of mice was disrupted. As consequence, the gut microbiota of these mice was altered compared to the control group. Green tea polyphenols administration promoted the relative abundance of *Bacteroidetes* while inhibited *Firmicutes*. However, these authors failed to prove the connection between gut microbiota, tea polyphenols, and circadian rhythm.

## 5. Conclusions

Green tea has been related to multiple health benefits and some of them are dependent on an inter-play with gut microbes. Tea polyphenols can be metabolized by gut bacteria, which seems to promote the growth of some beneficial species. On the other hand, polyphenols are also able to hinder the growth of some pathogenic bacteria, especially those of gram-positive. Green tea–gut microbiota inter-play seems to influence several tea-related bioactivities. Many of them are related to each other; an example is the relationship between obesity and lipid metabolism. Tea compounds are able to correct dysbiosis originated by high-fat diets and to influence the growth of some bacterial species involved in lipid or bile salts metabolism. In the case of inflammatory diseases and cancer, green tea reduces the production of pro-inflammatory substances (such as LPS either by inhibiting the growth of LPS-producing bacteria or by altering its secretion) and influences inflammatory pathways; usually green tea compounds have been shown to correct the microbial dysbiosis associated with these diseases. Therefore, metabolization of green tea compounds by gut microbes seems to be related with green tea health benefits, namely its antioxidant capacity, by breaking down parent phenolic compounds into smaller ones, and its significance in the cancer field in reducing inflammation, correcting dysbiosis, or controlling the production of harmful metabolites. It is also important to mention their effects on obesity, by promoting the growth of bacterial species related linked to lean people and correcting dysbiosis found in obese people, and in the lipid metabolism, by influencing the growth of bacteria with metabolic pathways involved in lipids and bile acids metabolization, and correcting dysbiosis resulting from high-fat diets. Moreover, they have also been related to inflammatory diseases by promoting the growth of beneficial bacterial species that regulate inflammatory pathways, and by reducing the secretion of proinflammatory substances such as LPS. Finally, in Parkinson’s disease, green tea EGCG seems to influence gene expression (totM gen) via gut microbiota. 

## Data Availability

Data sharing not applicable.
